# Engaging Communities to Develop and Sustain Comprehensive Wellness Policies: Louisiana’s Schools Putting Prevention to Work

**DOI:** 10.5888/pcd11.130149

**Published:** 2014-03-06

**Authors:** Elizabeth A. Gollub, Betty Monroe Kennedy, Brandi F. Bourgeois, Stephanie T. Broyles, Peter T. Katzmarzyk

**Affiliations:** Author Affiliations: Betty Monroe Kennedy, Stephanie T. Broyles, Peter T. Katzmarzyk, Pennington Biomedical Research Center, Baton Rouge, Louisiana; Brandi F. Bourgeois, Louisiana Tobacco Control Program, Louisiana Department of Health and Hospitals, Baton Rouge, Louisiana.

## Abstract

**Background:**

Tobacco use, obesity, and physical inactivity among Louisiana’s youth pose a serious public health problem. Given the potential of school environments to affect student well-being, the Louisiana Tobacco Control Program developed and tested a pilot program, Schools Putting Prevention to Work*.* The objective was to assist school districts in developing a comprehensive school wellness policy and engaging their school community to generate environments that support healthful choices and behaviors.

**Community Context:**

The pilot was implemented in 27 school districts, reaching an estimated 325,000 people across the state. Demographics of participating students were similar to all Louisiana’s public school students.

**Methods:**

A school wellness project state team advised project development. A subgroup that included contractors and partners implemented and modified the pilot. Sites were selected though an application process. Site representatives received trainings, technical assistance, and funding to organize school-based support-building activities and coordinate a school health advisory council to develop policy and sustain healthy school environments. Project sites reported progress monthly; evaluation included data from sites and project administrators.

**Outcome:**

Twenty-five comprehensive school wellness policies (covering 100% tobacco-free schools and daily physical activity and healthier cafeteria items) were approved by school boards. Environmental changes such as physical activity breaks, healthier vending options, and tobacco-free campuses were adopted.

**Interpretation:**

This pilot demonstrated a successful approach to achieving policy and environmental change. The state team engaged and guided school districts to motivate students, parents, faculty/staff/administration, and businesses to establish and maintain opportunities to improve lifestyle health.

## Background

Almost all long-term tobacco use begins during childhood and adolescence (1), as each day nearly 3,500 American youths try their first cigarette (2). In Louisiana, smoking rates and the use of smokeless tobacco among high school and middle school students exceed national averages by more than 50%. According to the 2011 Louisiana Youth Tobacco Survey conducted by the Louisiana Department of Health and Hospitals, more than 42% of Louisiana’s public school students reported that they were never taught about the dangers of smoking, and among Louisiana’s youth who smoke, approximately half (47%) reported that they want to quit. Youth tobacco behavior is a significant concern for the state.

The prevalence of overweight (19.5%) and obesity (16.1%) among Louisiana’s high school students is another serious concern (3). In addition to the links between body mass index and various chronic diseases/conditions, being overweight is associated with unhealthy weight-control behaviors (4). Among Louisiana’s high school students, approximately 36% reported that they tried to lose weight by fasting, vomiting, and using laxatives or over-the-counter diet aids (3). Furthermore, Louisiana’s youth are insufficiently physically active (5). In 2011, only 24% of Louisiana’s high school students met Louisiana Department of Health and Hospitals guidelines for aerobic physical activity (6); 42% had daily physical education classes; and 51% played on a school sports team at some point during the year (3). Recently, an examination of calorie intake among US youth noted that obese and overweight children consume fewer calories than do their healthy-weight counterparts, suggesting that energy expenditure/physical activity might be a particularly relevant component of weight management during adolescence (7).

Since the Women, Infants and Children (WIC) Reauthorization Act of 2004, which mandated the establishment of local school wellness policies, school districts have been struggling to develop and implement policies with guidelines for on-campus food and beverages, nutrition education, and physical activity (8). The importance of this Act lies in the potential of the school environment to affect the health and well-being of students (9). More recently, as a result of the Healthy Hunger-Free Kids Act of 2010, the US Department of Agriculture’s school meal guidelines have been revised to improve the quality of nutrition provided to our nation’s youth. Changes to the National School Lunch Program went into effect July 1, 2012 (10). The implementation of food, nutrition, and physical activity guidelines in schools is a recommended response to the increasing prevalence of obesity (11). School wellness policies are effective intervention points for managing youth tobacco behaviors as well (12,13).

The youth of Louisiana would benefit from schools that consistently provide healthy food choices, physical activity opportunities, and tobacco-free campuses (14–16). This was the impetus for the Louisiana Tobacco Control Program’s (LTCP’s) Schools Putting Prevention to Work (SPPW) pilot program. The pilot objective was twofold: to assist selected public school districts to develop a comprehensive school wellness policy and to engage the community (students/staff/parents/businesses) to support, implement, and sustain healthy behaviors by generating a school environment conducive to healthy choices and activities. SPPW was funded under the statewide policy and environmental change component of the Communities Putting Prevention to Work as a step toward reducing tobacco use and obesity among Louisiana’s youth.

## Community Context

The SPPW pilot was implemented in 27 of Louisiana’s 70 school districts across the state (Figure). It is estimated that the SPPW project reached 302,450 students and 22,080 teachers and staff. SPPW activities also reached parents and guardians and other community members. The racial and economic composition of SPPW students reflected that of the state’s public school students (17). Before the pilot, 23 of the 27 participating sites claimed to have a comprehensive wellness policy. However, a systematic review of policy components indicated that none of these policies met the SPPW criteria for a “comprehensive” policy, which is one that calls for 100% tobacco-free school campuses and activities, a minimum of 30 minutes per day of physical activity, and the availability of healthier food and beverage choices in the cafeteria and throughout the campus (18 ).

**Figure:**
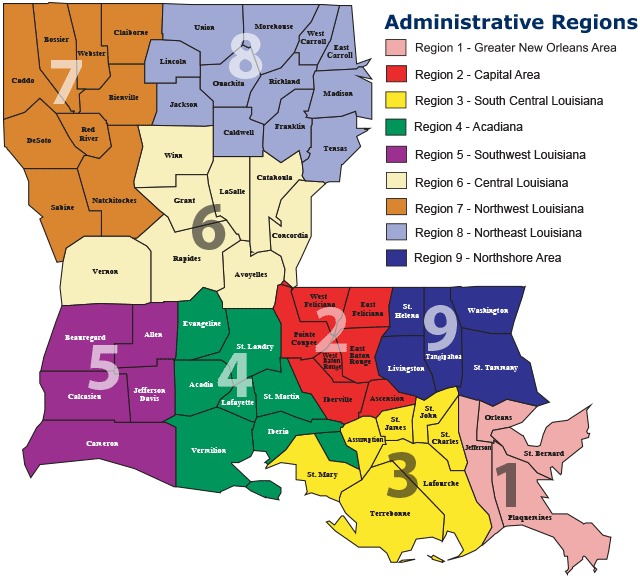
Schools Putting Prevention to Work: 27 site locations.

## Methods

The SPPW pilot consisted of a series of activities that began with an application/site selection process and ended with an approved comprehensive wellness policy. Participating school districts were required to 1) coordinate an active school health advisory council (SHAC) to assist with policy development and sustain implementation; 2) organize a local SPPW kick-off event to publicize the target issues; 3) implement a local media campaign to communicate messages about healthy eating, physical activity, and tobacco-free living to the school community; 4) develop and authorize a district-wide comprehensive wellness policy; and 5) plan for implementation and sustainability of the wellness policy. Participating districts were expected to implement their policy and continuously integrate successful wellness activities and modifications into classroom/school-based routines and practices. Guided by MAPPS (Media, Access, Point of decision information, Price, and Social support/services) interventions for CDC’s Communities Putting Prevention to Work, sites had the flexibility to develop plans and activities that were most appropriate for their community. Each participating site was given a $17,000 minigrant to defray the costs of project activities. Half of the grant funds were distributed at project initiation and half upon completion of the required activities. SPPW was carried out from June 2010 through January 2012.

### Administration

The LTCP maintained overall responsibility for this project but collaborated with the school wellness project state team (state team), a stakeholder advisory group organized for this purpose. The state team was made up of representatives from the Department of Health and Hospitals’ Chronic Disease Unit’s Tobacco Control Program, the Governor’s Council on Physical Fitness and Sports, the Department of Education’s Safe and Healthy Schools, the Louisiana Obesity Council, the Louisiana School Boards Association, Pennington Biomedical Research Center, LA Action for Healthy Kids, the Louisiana Public Health Institute, the University of Louisiana’s Picard Center, all of which have a statewide reach, and the Rapids Foundation, which serves 9 parishes in central Louisiana. The state team included experts in tobacco prevention, youth programming, physical activity education, nutrition and school food service, evaluation, school health and wellness, and policy analysis. These experts soon became the state team work group.

Daily project management was contracted with the Louisiana School Boards Association. A paid pilot coordinator functioned as project liaison, instructor, and comptroller, working closely with the LTCP, each project site, and the state team. The LTCP funded project evaluation through a contract with Pennington Biomedical Research Center. The external project evaluator participated in project planning, survey development/implementation, training events, and followed state team activities.

The LTCP used an application process to select project sites. For this purpose, the pilot coordinator and the state team work group drafted a detailed request for applications that was released on July 6 and distributed by mail and e-mail to school superintendents and school board presidents. The opportunity was announced at meetings of the Children’s Cabinet Advisory Board, the Louisiana Obesity Council, LaCapitale of the Links, Inc, and the annual Southern Obesity Summit. To further promote the opportunity, the Louisiana School Boards Association’s executive director, legislative liaisons, and school wellness coordinator telephoned key individuals around the state. A technical assistance conference call was held on July 22 to respond to questions and issues. Applications were due on August 6, 2010.

Applications were scored by the state team work group in accordance with a scoring rubric designed to favor the school districts that could benefit most from SPPW participation. The state team allocated priority points to districts that serve a largely at-risk demographic, are rural, did not have a functional SHAC, and did not have an adequate wellness policy. Points were also given for administrative capacities. The list of grant recipients was released as a statewide event, and contracts with the 27 school districts were formalized.

### Training

Each participating district selected a site coordinator to develop and manage SPPW activities. Each coordinator, generally with 1 or 2 additional project representatives, attended 3 day-long training sessions during the project period. Each training session was developed by the state team work group and evaluated to inform future program development and decision making.

The first training session was held at the beginning of the project period. It covered background information on the burden of tobacco and obesity and the role schools can play in food selection, physical activity, and tobacco-free environments. It also covered the project/contract deliverables, the *how-to* of developing a SHAC and district wellness policies, MAPPS strategies, action planning, and project evaluation. This training was held in both southern and northern Louisiana to accommodate all projects.

The second training session was held 6 months later. This session revisited the topics of action planning and MAPPS strategies because they were reported as problematic for several project sites. The session further covered the *how-to* of working with school boards and sustaining program achievements. Time was also devoted to the sharing of successes and lessons learned in a panel discussion led by selected site coordinators. Evaluation findings from the SPPW management perspective were shared.

The third training session was developed and scheduled as a wrap-up. The agenda included an overview of project accomplishments, presentations on district-specific changes resulting from the SPPW project, additional training on sustainability planning, and discussions on future school wellness activities and strategies.

The pilot coordinator monitored SPPW activities for compliance with project timelines and milestones through monthly progress reports submitted from each site. Site coordinators were encouraged to include newspaper articles, radio spots, and photos with their reports. Through a series of 3 open-ended surveys, the evaluator collected data from site coordinators on their experiences with processes such as SPPW message promotion and action planning, interacting with SHACs and school boards, problem solving, systematic reporting, and sustaining and increasing positive school-based practices and changes.

Similarly, the evaluator surveyed state team members on lessons learned during planning and implementation processes. All information was used to verify that project requirements were met, to describe how they were achieved, to assess their impact on students and community, and to inform program modifications.

## Outcome

The SPPW application window was a short 1-month period, but the opportunity was well publicized. It was acknowledged by 1 applicant as “the most publicized grant I have ever seen.” However, the timing of the application process was inconvenient. Several site coordinators reported that many district employees take vacation in July so the August deadline presented a challenge.

The SPPW engagement process began in March 2010 with the organization of the school wellness policy state team. This group of 18 stakeholders from around the state facilitated comprehensive project planning and shaping through routine quarterly meetings. By July, however, the state team work group emerged as a core of members actively involved in informing and guiding SPPW implementation. The pilot sites viewed this multidisciplinary management team as an asset to the project.

According to training evaluation reports, the first training session assisted site coordinators by clarifying how to use and convey information relevant for building support for SPPW and school-based change. The second training session addressed the coordinator’s policy presentation concerns, suggested new activities or ideas that could be replicated in local communities, and prompted thinking along new lines. Notably, site coordinators reported that as a result of this training, they felt prepared to approach their school boards with their wellness policies. The third training session provided the opportunity for site coordinators to present their project successes, lessons learned, and next steps. After the third training session, all participants reported that they were able to pinpoint project areas that still need work, use suggested approaches to problem solving, and adapt new ideas/activities to sustain their wellness efforts. An unexpected product of the training sessions was a list of items submitted by the site coordinators that prompted actions by the state team work group, ultimately strengthening the SPPW program (Table 1).

**Table 1:** Changes to SPPW Resulting From Site Coordinator Comments

Session	Item of Concern	State Team Work Group Response
1	Confusion about daily vs weekly physical activity recommendations for children	Develop document comparing federal guidelines to school-based recommendations
1	Confusion about application of MAPPS/action planning to project site objectives	Revise SPPW coordinator schedule to allocate time for individualized project site assistance
1	Interest in communication techniques for reaching specific groups (parents, teachers, students, school boards)	Arrange a presentation on this topic for the second training session
1	Interest in knowing what activities other SPPW pilot sites are doing as part of their project	Arrange an activities-to-date panel presentation from selected coordinators for second training session
2	Interest in learning about successful, replicable activities around the state	Develop a third training session to feature summary presentations from each project site; organize interactive discussion session on achieving sustainability
2	Requests for additional guidance on approaches to SPPW sustainability	
3	Need for assistance with local expansion of wellness agenda	Use information to inform planning for future technical assistance/trainings

Abbreviations: SPPW, Schools Putting Prevention to Work; MAPPS, Media, Access, Point of decision information, Price, and Social support/services.

Early in the project year, site coordinators established a new SHAC (20 sites) or revitalized an existing SHAC (7 sites), enlisting membership from the school district community. Sites reported that these current SHACs incorporated a more diverse representation, became more active and influential with school district policy, and began to rethink school health issues. Site coordinators worked with their SHAC to keep the school board and district administrators informed of SPPW activities as they progressed. To date, each SHAC remains in place and continues to promote a healthier school environment.

The kick-off event and a small-scale media campaign were in place to generate and reinforce interest in healthy food choices, physical activity, and tobacco-free environments among the school community. Together, these activities consisted of various forms of rallies; sporting events; programs and workshops; incentives; healthy food tasting; poster contests; and messaging through radio, television, billboards, newspapers, websites, flyers, and local celebrities. Data from progress reports and surveys indicated that 1 project developed their media campaign around billboards that were so hard to read that the campaign was ignored. But among the other sites, approximately half of the kick-off events and 92% of the media campaigns reached members of the entire intended target (the school community). Furthermore, 89% of these projects engaged the larger community through collaborations with foundations, universities, athletic associations, law enforcement, or other local agencies.

One project leader stated that the kick-off event was “unnecessary — that it just added more work” and noted that several parents resented the anti-smoking/tobacco information. But the other project leaders reported that the kick-off was a positive event that was interesting to attendees and prompted talk of behavioral change among students, parents, and administrators. Project leaders reported that the media campaign motivated some degree of healthier behaviors:

- Students would stop to show me the snack they brought from home and ask me if it is healthy.
- Students are telling [their parents] how to eat better and to buy healthier food.
- Some teachers are now [incorporating] exercise into their class routine.
- Several administrators and teachers are wearing their pedometers at school and closely watching their eating habits.
- Parents are volunteering and asking how they can help.

Of the original 27 pilot sites, 25 sites (36% of all school districts in the state) developed the comprehensive policy and received approval by their district school board. One site withdrew because of administrative complications; the other site drafted policy components but would not create a single comprehensive policy as required. In addition, each of the completing sites adopted various environmental changes that over time could improve health behaviors in the school communities (Table 2). Each of these sites also held sustainability discussions with their SHAC and/or district administrators, and 9 sites produced a sustainability plan. Site coordinators understood the value of maintaining an active SHAC and the benefit of continuously engaging the greater community to institutionalize healthful choices and normalize healthful behaviors.

**Table 2:** Summary of School-Environment Changes or Practices Adopted by SPPW Pilot Sites^a^

Type of Change	Description^b^	Number of Sites Adopting Change^c^^,^ d
**Vending**	Removal of soft-drink machines; providing only juice and water as beverages; snack vending items must be on Louisiana’s Approved Vending List for Schools (18); addition of healthy items that are <150 calories.	3
**Snacking**	Healthier snack selections; no candy; fruits/vegetables several times per week; healthy refreshments at school board meetings and assemblies; list (for parents) of acceptable classroom snacks/party foods.	8
**Cafeteria**	Meals made with more whole grains; more fresh fruits and vegetables; foods are baked (not fried); use leaner meats; no sugary breakfast foods; pre-plated salad; salad bars; new items on menus.	8
**Wellness programs**	Schoolwide tasting days; informational programs/local media for students and for community; annual wellness events; parent newsletters with practical nutrition/fitness tips.	11
**Fitness activities**	Student fitness events; 15 min/d of outside play; organized sports/games at recess; “field time” replacing class parties; year-long lap-running competition; other contests; walking programs; extracurricular sports programs; pedometer distribution; classroom activity breaks; new physical activity equipment; integrated fitness/academic curriculum.	16
**Rewards and fundraisers**	Nonfood items for fundraising; nonfood/noncandy-based reward systems.	2
**Tobacco prevention**	Tobacco-free signage; all school-related events and activities are tobacco-free; cessation programs; Kick Butts participation; fax-to-quit program.	17
**Teacher training**	Fitnessgram; Wel-Net; new games; new physical activity equipment.	5

Abbreviation: SPPW, Schools Putting Prevention to Work.

a Data are based on survey responses from 21 of the 25 SPPW sites completing the pilot.

b Changes/practices are “as reported” by site coordinators; they are not standardized across sites.

c Sites could have adopted any or several components described in the category, at any or all of the schools in the district.

d The number of changes per site ranged from 0 to 4 with a median of 2.

Each site coordinator dealt with implementation challenges. Several of these challenges and coping strategies were common to more than 1 site. First, site coordinators reported that early on their efforts were frequently interrupted by holidays and weather-related school closings. Though frustrated, they persevered. Next, most of the coordinators indicated that meetings, record keeping, and monthly reporting responsibilities created major scheduling stresses; they would have preferred quarterly reporting. However, they worked through it by prioritizing and applying other time-management strategies. A larger issue was that some school principals and staff assigned to project activities viewed their SHAC and the project as irrelevant. When necessary, site coordinators circumvented these principals by working through assistant superintendents; when possible, coordinators replaced uninterested staff. Several other site coordinators reported that their SHAC was disjointed and struggling. After consulting with successful SHACs on best practices, these groups began to focus on team building and leadership.

## Interpretation

The SPPW pilot demonstrated a unique approach to achieving its objectives. It is the first program to partner with a school boards association, to make use of a state team advisory group to provide guidance and training to site coordinators on tobacco and obesity strategies simultaneously, and to create incentives for policy development. By the end of the pilot period, the 25 completing sites had achieved the community buy-in that is essential for implementing new wellness policies, reshaping their school environment, and sustaining the effort. A 1-year follow-up on SPPW-related efforts and activities is in progress. Over the long term, the Louisiana Youth Tobacco Survey and the Youth Risk Behavioral Survey can be used to monitor trends in key food, tobacco use, and physical activity habits of Louisiana students.

Lessons Learned

1. For SPPW to comfortably coincide with the academic year, project planning and organization should be in place before summer recess.
2. Working through a large statewide group of stakeholders was constructive for project initiation. Once the pilot was in place, a smaller workgroup of key stakeholders was more effective and efficient.
3. Training for site coordinators is important for project success. Participants reported that the trainings provided guidance and focus, facilitated collaborations, and fostered confidence.
4. Most of the participating pilot sites reported that they could not have achieved their successes without the associated minigrant. Though minimal, the funds served as a goodwill gesture and helped administrators justify staff time and effort from among competing activities.
5. As a rule, SPPW activities piqued the interest and prompted participation of students, encouraged parental involvement, and initiated teacher-driven classroom and school-based changes. However, some SPPW changes (eg, a switch to whole-grain items in cafeterias; 100% tobacco-free sporting events) required time and repetition before they became acceptable.
6. Project support among SHACs, administrators, and school boards was built and maintained through straightforward, ongoing communications. This support facilitated the process of wellness policy passage by each district’s school board.

The Louisiana SPPW model is an effective approach to establishing school wellness policies and healthier school environments.
